# Exploring the Causal Effects of Micronutrient Supplementation on Susceptibility to Viral Pneumonia: A Mendelian Randomization Study

**DOI:** 10.3390/pathogens14030263

**Published:** 2025-03-07

**Authors:** Shunran Li, Mingting Cui, Ziwen Song, Jianhui Yuan, Caijun Sun

**Affiliations:** 1 School of Public Health (Shenzhen), Sun Yat-sen University, Shenzhen 518107, China; lishr53@mail2.sysu.edu.cn (S.L.); cuimt@mail2.sysu.edu.cn (M.C.); songzw@mail2.sysu.edu.cn (Z.S.); 2 Shenzhen Key Laboratory of Pathogenic Microbes and Biosafety, Shenzhen Campus of Sun Yat-sen University, Shenzhen 518107, China; 3 Nanshan District Center for Disease Control and Prevention, Shenzhen 518054, China; 4 Key Laboratory of Tropical Disease Control (Sun Yat-sen University), Ministry of Education, Guangzhou 514400, China; 5 State Key Laboratory of Anti-Infective Drug Discovery and Development, School of Pharmaceutical Sciences, Sun Yat-sen University, Guangzhou 510006, China

**Keywords:** viral pneumonia, micronutrients, vitamins, minerals, mendelian randomization study

## Abstract

Viral infections have been a severe challenge for global public health, and viral pneumonia is becoming increasingly critical in the post-pandemic era. Observational and basic studies have demonstrated a strong link between host nutrient status and anti-viral immune responses, and nutritional supplements were shown to improve the prognosis of viral infectious diseases. However, there is limited research on the relationship between essential micronutrients and the susceptibility to viral pneumonia. In addition, current studies are often confounded by biases and reverse causality, undermining their reliability. In this study, to fill the gap, we employed Mendelian randomization to investigate the causal relationship between supplementation of vitamins and minerals and the susceptibility to viral pneumonia. Our analysis found that vitamin B6 is a protective factor against viral pneumonia, while selenium supplementation is a risk factor. These findings provide insights for the use of dietary supplements and the prevention and control of viral pneumonia, especially when micronutrient supplementation is used as an adjunctive therapy for viral infections.

## 1. Introduction

The frequent emergence of viral infectious diseases poses a significant threat to public health. Pneumonia remains a leading cause of hospitalization and death and is also one of the top causes of disability-adjusted life years (DALYs) globally [1,2]. Approximately 1 million people over the age of 65 have community-acquired pneumonia (CAP) in the US per year [3], and respiratory viruses are identified in approximately 25% of CAP patients [4]. A study in Bangladesh found that among 1248 pneumonia visits with laboratory testing, 803 (64%) were infected with viral pathogens [5]. And increasing studies suggest that the etiology of pneumonia is more frequently due to viral infections instead of bacterial infections [6]. Viral pneumonia is usually caused by influenza, parainfluenza, respiratory syncytial virus (RSV), human metapneumovirus, adenovirus, and SARS-CoV-2, which is an important cause of morbidity and mortality among young children (<5 years) and aging adults >50 years [6], as the SARS-CoV-2 pandemic has emphasized.

Currently, the primary strategy for the prevention and control of viral pneumonia is vaccination. However, vaccination only protects against a few viruses and exhibits limited efficacy. Additionally, antiviral drug resistance is becoming an increasingly serious issue.

As a result, it is of great importance to develop new strategies against viral pneumonia. Increasing studies have shown that in-depth investigation on the relationship between host cell metabolism and antiviral immunity may reveal novel targets for the development of antiviral agents [7,8,9,10,11,12]. Interestingly, it is recognized that the nutritional status of the hosts is also vital in the defense of infectious diseases, as comprehensively reviewed [13,14,15]. Multiple micronutrients (vitamins and minerals) play crucial roles in supporting the immune system. People with low intakes or deficient in these micronutrients show immune impairments and increased susceptibility to infectious diseases, especially respiratory and gastrointestinal diseases [16,17]. A meta-analysis of seven systematic reviews showed strong evidence that vitamin D supplementation reduces the risk of mortality (odds ratio: 0.48, 95% CI: 0.346–0.664; *p* < 0.001) in COVID-19 patients [18]. Zhang et al. found that oral administration of vitamin A derivatives in yellow fever virus-infected individuals significantly reduced the likelihood of mosquitoes feeding on their blood and acquiring the virus, thereby preventing the efficient establishment of the “host–mosquito” transmission cycle in the natural environment [19]. Despite these fundamental and observational studies, evidence of the link between some of these micronutrients and viral pneumonia at the population level is still missing. And the existing observational studies suffer from the risk of confounding factors and reverse causality. Thus, exploring the causal relationship between various micronutrients and human resistance to viral pneumonia is still needed, which could guide the prevention and control of the disease and potentially help to identify safe and effective antiviral strategies.

Mendelian Randomization (MR) is an application of instrumental variable analysis which uses genetic variation as a natural experiment to improve causal inferences from observational data of large-scale genome-wide association studies [20]. The principle of MR refers to Mendel’s second law of independent segregation of genetic alleles when DNA is transmitted from parents to offspring at gamete formation [20]. In this process, the alleles of the parental genotype are randomly allocated to the offspring, similar to the random assignment of treatment in a randomized clinical trial (RCT), which is the gold standard for causal inference because it ensures that confounding factors are evenly distributed across the groups. By taking advantage of this natural randomization process, an MR study “randomizes” the participants by genetic variants associated with the exposure and thus reduces the risk of confounding. MR analysis allows for use of observational data to perform causal inference and thus benefits the studies of exposures which are not appropriate as an intervention.

This study aims to investigate the causal effects of vitamin and mineral intake on susceptibility to viral pneumonia using MR analysis, providing insights and perspectives for the prevention and treatment of viral pneumonia and also helping to understand the indispensable role of micronutrients on human health.

## 2. Materials and Methods

### 2.1. Data Source

The GWAS summary datasets of the exposure data were obtained from the UK Biobank [21]. The UK Biobank is a large-scale biomedical database and research resource containing de-identified genetic, lifestyle and health information and biological samples from half a million UK participants. The detailed information about the exposure dataset used in this study is listed in Table S1. Summary statistics data for viral pneumonia were retrieved from the FinnGen [22]. The FinnGen study is a large-scale genomics initiative that has analyzed over 500,000 Finnish biobank samples and correlated genetic variation with health data to understand disease mechanisms and predispositions. The project is a collaboration between research organisations and biobanks within Finland and international industry partners. According to the endpoint definition of FinnGen, the phenotype of viral pneumonia was identified by filtering the “Hospital discharge” and “Cause of death” related to viral pneumonia characterized by ICD 8, 9 and 10 [23]. These summary data contain 5446 cases, including 2555 females and 2891 males, and 42,276 controls, including 19,026 females and 22,987 males. The age distribution of the cases is primarily concentrated in the under-10 and over-65 age groups.

### 2.2. Genetic Variants Selection Criteria

The three core assumptions that must hold for valid results in an MR analysis include: (i) reliably associate with the risk factor under investigation (relevance assumption); (ii) not be associated with any known or unknown confounding factors (independence assumption); and (iii) influence the outcome only through the risk factor and not through any direct causal pathway (exclusion restriction assumption). To fulfil these assumptions, genetic variants achieving genome-wide significance with the exposures were primarily selected as instrumental variables (IVs) at the *p*-value cutoff of 1 × 10^−6^. The F statistic for these SNP was calculated using the following formula: Beta^2^/SE^2^. Only SNPs with F statistics large enough (F > 10) were considered to be strongly correlated with exposure, and thus the results of the MR analysis could avoid being affected by weak-tool bias [24]. In addition, to satisfy assumptions i and ii, we searched the SNPs already obtained through LDtrait {LDlink|An Interactive Web Tool for Exploring Linkage Disequilibrium in Population Groups, https://ldlink.nih.gov/?tab=home (accessed on 25 December 2024)} to find potential confounders and bypassing that might influence the association between vitamins and minerals with viral pneumonia [25]. SNPs associated with confounders were removed. Then, SNPs in high linkage disequilibrium (r^2^ > 0.001) were clumped in order to make sure the instrumental variables were independent. SNPs associated with the outcome were also excluded. In the case that SNPs strongly correlated with exposure were missing in the outcome dataset, we removed these SNPs in the next analysis. Harmonization was performed to exclude palindromic and incompatible SNPs.

### 2.3. Statistical Analysis

MR estimates, like inverse variance weighted (IVW), MR-egger and weighted median, were used in this study to evaluate the casual effects between exposures and the outcome. Sensitivity analysis, like Cochran Q test, Egger-intercept test, and leave-one-out analysis, were then performed, and heterogeneity of the instrumental variables were evaluated by applying Cochran’s Q test. Cochran’s Q test includes the MR-egger method and inverse variance-weighted method. Significant heterogeneity was defined as a *p*-value less than 0.05. Scatter plots and funnel plots were drawn to visualize the data. All statistical analysis were performed by using the TwoSampleMR R package (version 0.6.8).

## 3. Results

**Figure 1:**
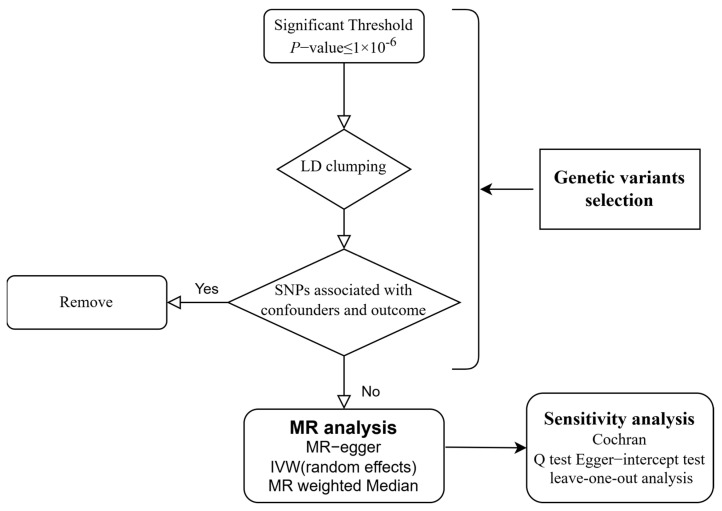
Flow chart of the study design. LD: Linkage Disequilibrium; SNP: Single Nucleotide Polymorphism; MR: Mendelian Randomization; IVW: Inverse Variance-Weighted.

This study is designed according to the STROBE-MR guidelines to avoid potential bias [26]. Our study design is shown in Figure 1. The causality between supplementation of micronutrients and susceptibility of viral pneumonia was explored by MR analysis for the first time.

**Figure 2:**
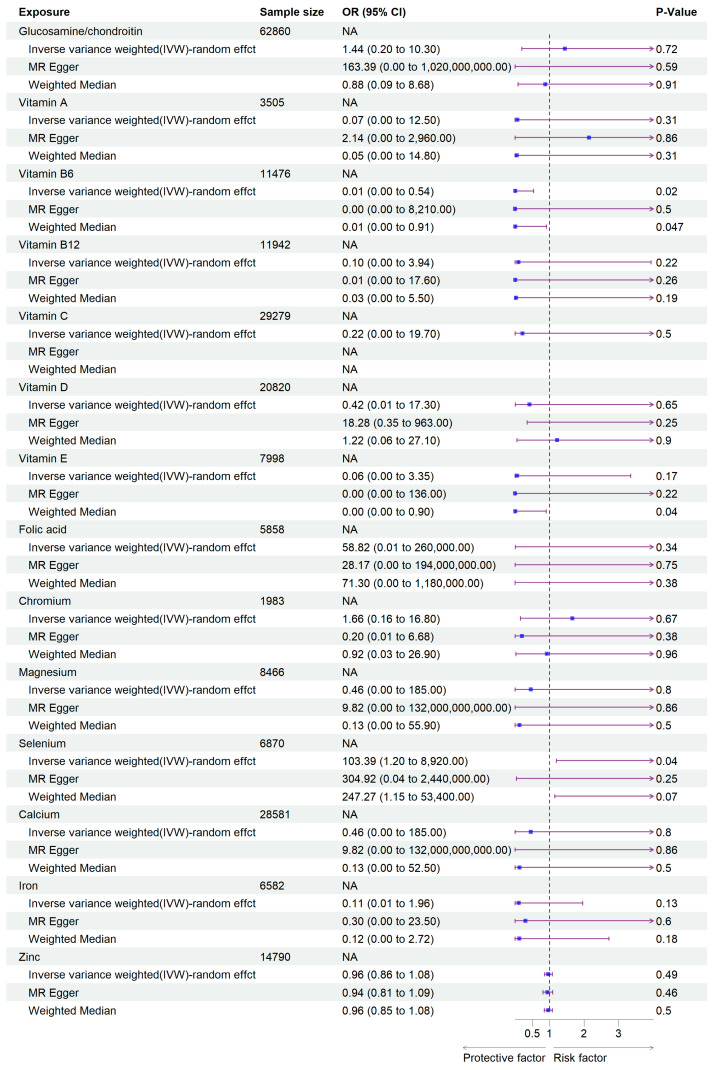
Forest plot of the MR analysis results. The purple arrow indicates the extension of the confidence interval; due to space constraints in the plot, the endpoints of the confidence interval cannot be displayed.

In this study, we extracted SNPs significantly related to the exposures according to the genetic variants selection criteria that we gave earlier. SNPs related to supplement use of Glucosamine/chondroitin, Vitamin A, Vitamin B6, Vitamin B12, Vitamin C, Vitamin D, Vitamin E, Folic acid, Chromium, Selenium, Magnesium, Calcium, Iron, and Zinc were selected. Detailed information of the screening process is presented in Table S2. In the MR analysis studying the association between vitamins and viral pneumonia susceptibility, we found that Vitamin B6 significantly reduced the incidence of viral pneumonia (95% CI: 2.74 × 10^−4^, 0.54; *p* = 0.023). However, the results showed that the supplementation of Glucosamine/chondroitin, Vitamins A, B12, C, D, E, and Folic acid had no significant influence on viral pneumonia, with ORs (odds ratio) 1.44 (95% CI: 2 × 10^−1^, 10.3; Glucosamine/chondroitin), 0.07 (95% CI: 3.50 × 10^−4^, 12.5; Vitamin A), 0.10 (95% CI: 2.51 × 10^−3^, 3.94; Vitamin B12), 0.22 (95% CI: 2.38 × 10^−3^, 19.68; Vitamin C), 0.42 (95% CI: 0.01, 17.2; Vitamin D), 0.06 (95% CI: 1.14 × 10^−3^, 3.35; Vitamin E), 58.81 (95% CI: 1.33 × 10^−2^, 2.60 × 10^5^; Folic acid) respectively. As for mineral intake, Selenium was found to be a risk factor against viral pneumonia (95% CI: 1.20, 8.92 × 10^3^; *p* = 0.04). Other minerals including Chromium, Magnesium, Calcium, Iron, and Zinc had no significant effect on the susceptibility of viral pneumonia. The ORs are 1.66 (95% CI: 0.16, 16.80; Chromium), 0.46 (95% CI: 1.14 × 10^−3^, 1.85 × 10^2^; Magnesium), 0.46 (95% CI: 1.14 × 10^−3^, 1.85 × 10^2^; Calcium), 0.11 (95% CI: 5.79 × 10^−3^, 1.96, Iron), and 0.96 (95% CI: 0.85, 1.08; Zinc), respectively (Figure 2, Table 1 and Table 2).

**Figure 3:**
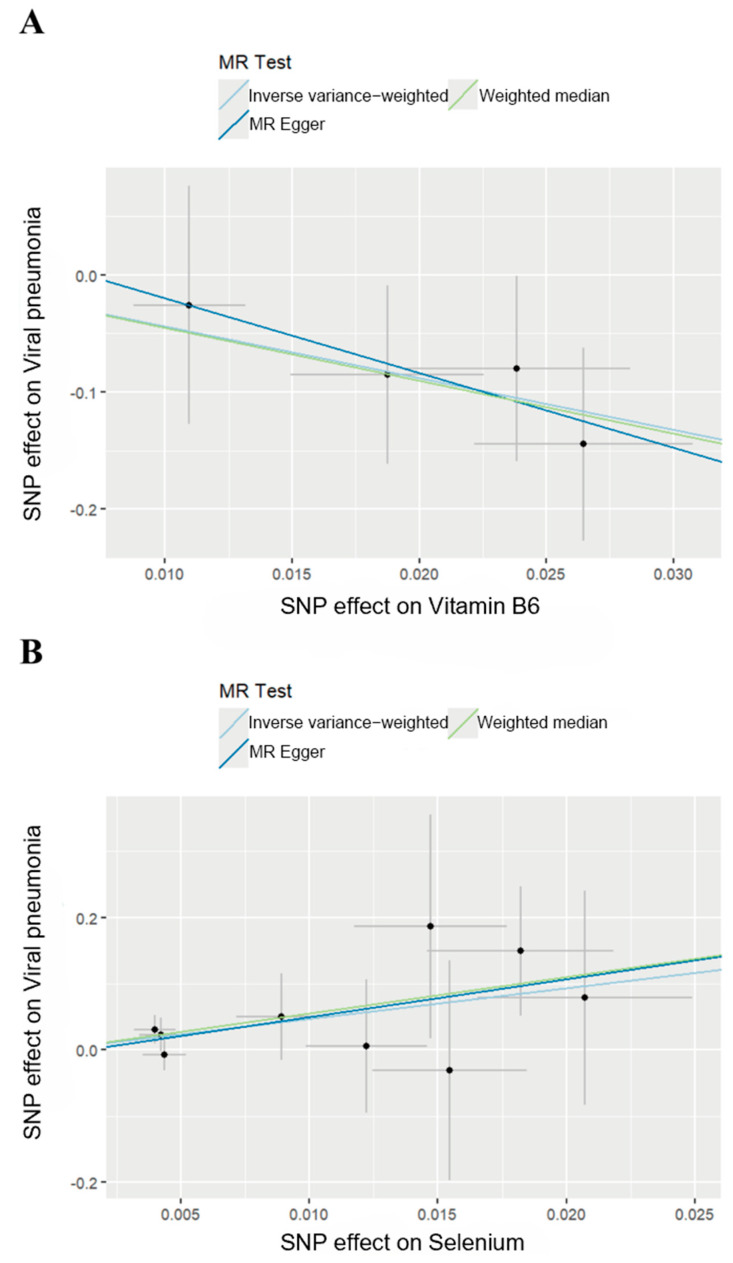
Scatter plots of MR analysis. (**A**) Scatter plot of effect of Vitamin B6 on viral pneumonia. (**B**) Scatter plot of effect of Selenium on viral pneumonia.

**Figure 4:**
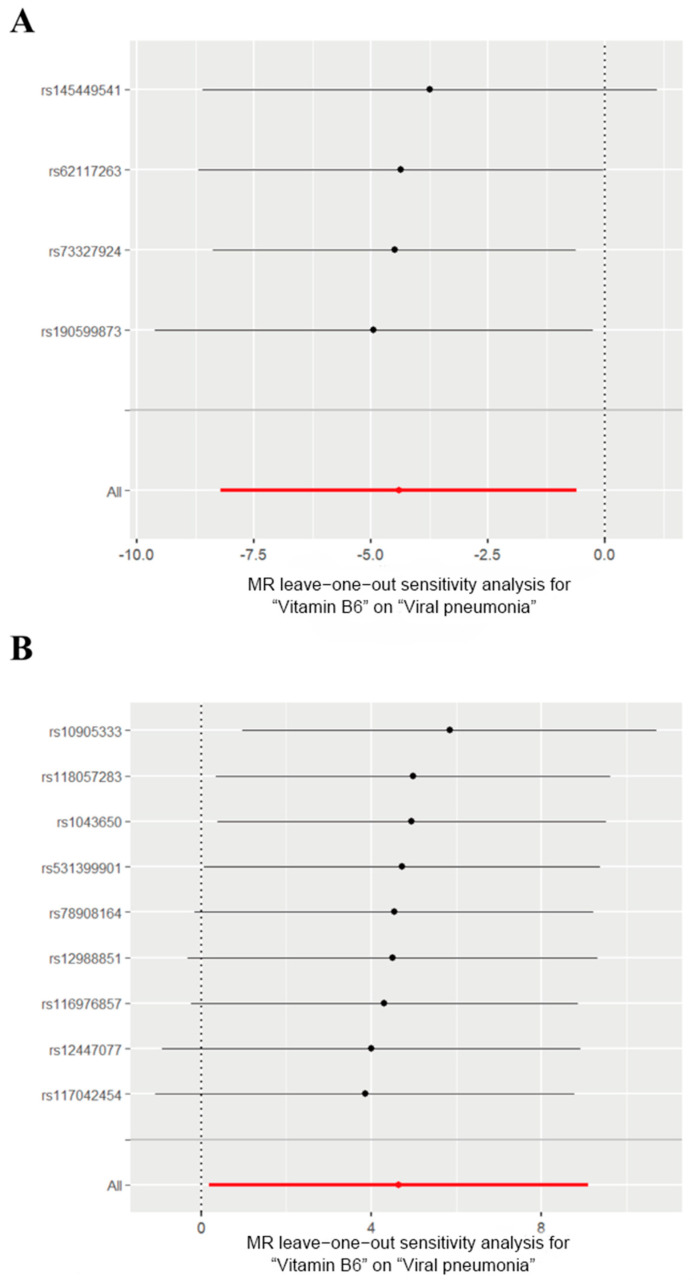
Leave-one-out analysis of the effects of Vitamin B6 and Selenium on viral pneumonia. (**A**) Leave-one-out analysis of the effects of Vitamin B6 on viral pneumonia. (**B**) Leave-one-out analysis for the effect of Selenium on viral pneumonia. The red lines represent the cumulative effect of all SNPs.

We then generated scatter plots from the MR analysis of Vitamin B6 and Selenium with the outcome to examine these positive results more closely (Figure 3). After that, a series of analysis was conducted to test the robustness of these results. Leave-one-out analysis was applied to evaluate the sensitivity of the MR analysis (Figure 4). Although the removal of rs145449541 led to an insignificant result in the MR analysis of Vitamin B6 on viral pneumonia, the direction of the effect remained unchanged. A similar situation was observed in the analysis of Selenium. Given the small number of nsnp, we considered the MR findings relatively robust.

In order to avoid potential bias, we used Egger Intercept to evaluate the pleiotropy, and no group in the MR test showed pleiotropy (Table S3). We also used Cochran’s Q test to perform the heterogeneity analysis, and SNPs related to Vitamin B6 or Selenium showed no heterogeneity (Table S3). Furthermore, the funnel plots showed no significant biases in SNPs selected for MR analysis (Figure S1). Together, we believed our results to be solid.

In conclusion, our results demonstrate significant causal effects of Vitamin B6 on reducing the susceptibility to viral pneumonia, and selenium was identified as a risk factor of viral pneumonia.

## 4. Discussion

In this MR Study, we explored the causal effects of 14 micronutrients on susceptibility to viral pneumonia. We identified for the first time that Vitamin B6 had a protective effect on the incidence of viral pneumonia, while selenium supplementation was a risk factor at the population level. These findings can provide valuable insights to facilitate future studies to develop better strategies for preventing viral pneumonia and improve our understanding of the complex role micronutrients play in anti-viral immunity, especially when micronutrients supplementation is used as an adjunctive therapy for patients infected with HIV and tuberculosis.

In 1949, Mirick et al. found that mice fed with diets deficient in Vitamin B6 for 8 days or longer before the inoculation of the Pneumonia Virus of Mice (PVM) were more susceptible to infection than control mice fed with complete diets [27]. After that, fundamental research revealed that Vitamin B6 is vital for the immune function in both human and animal models [28]. In clinical practice, Vitamin B6 is often used in combination with other medications to treat HIV patients with vitamin deficiency and improve the immune function of patients with chronic infectious diseases like tuberculosis. Although several studies suggested the potential antiviral function of Vitamin B6 [29,30,31,32,33], there were no studies at the population level to clarify the casual effects of Vitamin B6 on susceptibility to viral pneumonia. This study filled the gap and thus provided a better understanding of the biology of Vitamin B6 and the prevention of viral pneumonia.

In addition, other studies demonstrated the importance of other vitamins mentioned in this study on immune function. Among them, Vitamin D has received the most attention. It is reported that Vitamin A and D were associated with tuberculosis in HIV-infected patients [34], and Vitamin D in serum could be used to predict the antiviral response in chronic hepatitis C patients, suggesting an association between these vitamins and viral infectious diseases. Mechanistically, Vitamin D pre- and post-treatment could suppress the inflammatory response to influenza A (H1N1) infection in human lung A549 epithelial cells [35]. However, our study did not find significant causal relationships between Vitamin A, Vitamin B12, Vitamin C, Vitamin D, Vitamin E, and Folic acid with viral pneumonia. The role of these vitamins in viral infectious diseases requires further investigation.

Selenium is an essential trace element for mammalian redox biology [36]. Human and animal studies have shown that selenium status is a key determinant of the host response to viral infections [37]. It is now recognized that the selenium nutritional status of the host plays a leading role in the defence against infectious diseases [36]. Most studies suggest that selenium is a protective factor that prevents hosts from severe viral infection. For instance, Beck et al. found that selenium deficiency increased the pathology of influenza viral infection in a mouse model [38]. The expression of interferon-γ, a cytokine vital to both innate and adaptive immunity, greatly decreased in the selenium-deficient animal [39]. Mice that were fed the diet deficient in selenium developed myocarditis after infection of coxsackievirus B3 (CVB3), CVB3/0 [40]. In addition, some research indicated that selenium could be used as a potential agent to treat viral infections [41,42]. These studies were conducted in cell lines or animal models. At the population level, dietary supplementation to provide an adequate or supranutritional selenium supply has been proposed to confer health benefits for patients suffering from some viral diseases, most notably with respect to HIV and influenza A virus (IAV) infections [43]. However, our study showed a conflicting result, indicating that selenium supplement is a risk factor of viral pneumonia. This could be attributed to several reasons. First, most of these studies were performed on animal models. The difference in biology between mouse and human may lead to contradictory results. Second, although evidence at the population level showed that selenium had a protective effect on IAV infections, the summary dataset of viral pneumonia we used in this study did not include influenza infection, which may help to explain. In addition, we believe the contradicted inference could also be attributed to the difference in the study design and participants. Our study is a Mendelian randomization study that applied GWAS summary data of a wide population range, while the study indicating selenium could protect participants from IAV infection was performed in a population that received IAV vaccination [44]. The protective effect of selenium could be ascribed to the improvement of the antibody titre against IAV after vaccination. No evidence showed that selenium itself could protect people from IAV infection. This conflict should be a reminder to be more careful about selenium supplements for therapeutic use.

Viral pneumonia is predominant in young children [6]. And due to the physiological variance between children and adults, micronutrient supplementation would possibly exert different functions. Thus, figuring out the effect of micronutrients on viral pneumonia among this specific group is essential. Existing research has reported the association between vitamins and viral pneumonia, especially COVID-19 in young patients. Multiple observational studies have shown that Vitamin D levels in children with COVID-19 are significantly lower than in healthy groups [45,46,47,48,49,50], and lower 25 OH vitamin D levels were associated with higher inflammation markers and a more critical clinical condition [48,51,52,53], which means vitamin D deficiency is a risk factor for COVID-19 [47,54,55,56,57,58,59].

Meanwhile, Vitamin D_3_ supplements (1200 IU/d) could significantly lower the incidence of seasonal influenza A in schoolchildren (18.6% of the placebo group compared to 10.8% of the treatment group), according to a randomized trial conducted in 2008 [60]. However, our study did not find a significant relationship between vitamin D and viral pneumonia. Possible reasons could be the different distribution of age, sex, or other confounding factors in the study population, the limited power of our analysis, and the different outcomes we chose to focus on. Also, the association between vitamin D status and COVID-19 that current studies show could be confounded by reverse causality. This conflict suggests to apply the negative results in this study more carefully.

As for trace elements, Zinc levels in COVID-19-positive children were found to be lower than in the healthy control group [61]. A large-scale meta-analysis suggested zinc supplementation held a protective effect for viral respiratory tract infections (including COVID-19) in Asian children and not in other regions [62]. Since our genetic analysis was restricted to individuals of European ancestry, the findings of this study may not be generalizable to non-European populations. Future studies that include diverse populations would be needed to validate these results.

In fact, only less than 6% of COVID-19 cases occurred in children, and most of those cases involved minor symptoms similar to other respiratory infections, the biological explanation for this still remaining elusive [63,64]. Our study might provide a new perspective that the different micronutrient statuses of children and adults may contribute to it. A further stratified analysis would address this question and shed light on the complex function of nutrients in human health.

However, this study has several limitations. First, the dataset about vitamin and mineral supplement use was collected through a questionnaire and self-reported, which may introduce reporting bias. Additionally, although we knew that the route of administration was oral, the data on other detailed information, like the duration, dose and basal value of the micronutrients the participants administered, was not demonstrated. Thus, our study only reached the conclusion that Vitamin B6 supplementation is a protective factor of viral pneumonia and selenium is a risk factor. There is no evidence about what dose and how long we should take vitamin supplements. The potential heterogeneity of the intake of micronutrients of participants may also influence the result and weaken the statistical power of this study. Secondly, the GWAS summary dataset we used to select the genetic variants related to micronutrients intake was based on the UK population. Then, these genetic variants were utilized on a Finnish population to see if they were related to viral pneumonia infections. The heterogeneity of these two populations may introduce confounding bias. In addition, our GWAS summary data was from the European population, so the results may not be applicable to other populations with different genetic backgrounds. Meanwhile, we only investigated the causal effects of micronutrients on the susceptibility to viral pneumonia rather than severity or prognosis of it.

Together, this Mendelian randomization study provides insight to understand the biological effects of micronutrients and contribute to the control of viral infectious diseases. In the two inferences of the study, the protective effect of vitamin B6 against viral pneumonia is consistent with previous research showing that vitamin B6 benefits immune function and improves disease prognosis. However, our study also found that selenium may promote the development of viral pneumonia, which contrasts with the prevailing view that selenium’s antioxidant properties enhance immunity. Our findings suggest that the use of selenium supplements should be approached with caution.

## Figures and Tables

**Table 1:** Causal effects of vitamins and minerals on viral pneumonia–IVW and MR Egger.

GWAS ID	Exposure	Outcome	Inverse Variance Weighted		MR Egger	
			OR (95%CI)	*p*-Value	OR (95%CI)	*p*-Value
20084_473	Glucosamine/chondroitin	Viral Pneumonia	1.44 (0.20, 10.304)	0.72	163.39 (2.62 × 10^−5^, 1.02 × 10^9^)	0.59
20084_475	Vitamin A		0.07 (3.49 × 10^−4^, 12.49)	0.31	2.14 (1.55 × 10^−3^, 2.96 × 10^3^)	0.86
20084_476	Vitamin B6		0.01 (2.74 × 10^−4^, 0.54)	0.02	1.72 × 10^−3^ (3.59 × 10^−10^, 8.21 × 10^3^)	0.5
20084_477	Vitamin B12		0.10 (2.51 × 10^−3^, 3.94)	0.22	5.48 × 10^−3^ (1.70 × 10^−6^, 17.62)	0.26
20084_478	Vitamin C		0.22 (2.38 × 10^−3^, 19.68)	0.5	NA *	NA
20084_479	Vitamin D		0.42 (0.01, 17.25)	0.65	18.28 (0.35, 962.57)	0.25
20084_480	Vitamin E		0.06 (1.14 × 10^−3^, 3.35)	0.17	1.14×10^−5^ (9.55 × 10^−13^, 135.68)	0.22
20084_481	Folic acid		58.82 (1.33 × 10^−2^, 2.60 × 10^5^)	0.34	28.17 (4.08 × 10^−6^, 1.94 × 10^8^)	0.75
20084_482	Chromium		1.66 (0.16, 16.80)	0.67	0.20 (5.90 × 10^−3^, 6.68)	0.38
20084_483	Magnesium		0.46 (1.14 × 10^−3^, 1.85 × 10^2^)	0.8	9.82 (7.30 × 10^−10^, 1.32 × 10^11^)	0.86
20084_484	Selenium		103.389 (1.20, 8.92 × 10^3^)	0.04	304.92 (0.04, 2.44 × 10^6^)	0.25
20084_485	Calcium		0.46 (1.14 × 10^−3^, 1.85 × 10^2^)	0.8	9.82 (7.30 × 10^−10^, 1.32 × 10^11^)	0.86
20084_486	Iron		0.11 (5.79 × 10^−3^, 1.96)	0.13	0.30 (3.74 × 10^−3^, 23.48)	0.6
20084_487	Zinc		0.96 (0.86, 1.08)	0.49	0.94 (0.81, 1.09)	0.46

* NA: not available. When the number of SNPs ≤ 2, we used IVW only to conduct the MR analysis.

**Table 2:** Causal effects of vitamins and minerals on viral pneumonia–Weighted Median.

GWAS ID	Exposure	Outcome	Weighted Median	
			OR (95%CI)	*p*-Value
20084_473	Glucosamine/chondroitin	Viral Pneumonia	0.88 (8.93 × 10^2^, 8.68)	0.91
20084_475	Vitamin A		0.05 (1.59 × 10^−4^, 14.81)	0.31
20084_476	Vitamin B6		0.01 (1.32 × 10^−4^, 0.91)	0.047
20084_477	Vitamin B12		0.03 (8.84 × 10^−5^, 5.50)	0.19
20084_478	Vitamin C		NA *	NA
20084_479	Vitamin D		1.22 (0.06, 27.13)	0.9
20084_480	Vitamin E		4.10 × 10^−3^ (1.86 × 10^−5^, 0.90)	0.04
20084_481	Folic acid		71.3 (4.30 × 10^−3^, 1.18 × 10^6^)	0.38
20084_482	Chromium		0.92 (0.03, 26.90)	0.96
20084_483	Magnesium		0.13 (2.91 × 10^−4^, 55.91)	0.5
20084_484	selenium		247.27 (1.15, 5.34 × 10^−4^)	0.07
20084_485	Calcium		0.13 (3.10 × 10^−4^, 52.48)	0.5
20084_486	Iron		0.12 (5.00 × 10^−3^, 2.72)	0.18
20084_487	Zinc		0.96 (0.85, 1.08)	0.5

* NA: not available. When the number of SNPs ≤ 2, we used IVW only to conduct the MR analysis.

## Data Availability

The GWAS summary datasets related to vitamins and minerals are accessible under application at https://www.ukbiobank.ac.uk (accessed on 28 November 2024). The dataset involving viral pneumonia is accessible at Finngen [https://www.finngen.fi/en (accessed on 27 November 2024)]. FinnGen GWAS summary statistics can be downloaded from Google Cloud storage free of charge.

## Supplementary Materials

The following supporting information can be downloaded at: https://www.mdpi.com/article/10.3390/pathogens14030263/s1, Table S1: Detailed information of the GWAS summary dataset. Table S2: Detailed information of SNP selection. Table S3: Information for the Pleiotropy and Heterogeneity tests. Figure S1: Funnel plots of SNPs selected for MR analysis of Vitamin B6 and Selenium on viral pneumonia.
