# Supplementary material for: Exploring the Causal Effects of Micronutrient Supplementation on Susceptibility to Viral Pneumonia: A Mendelian Randomization Study
Source: Pathogens. 2025 Mar 7;14(3):263. doi: 10.3390/pathogens14030263 (PMC11944707; doi:10.3390/pathogens14030263)
Supplement: Supplementary file 1 [file pathogens-14-00263-s001.zip › pathogens-3461063-supplementary.pdf]

Table S1. Detailed information of the GWAS summary dataset.

GWAS ID	Exposure	Data showcase link
20084_473	Glucosamine/ chondroitin	http://biobank.ctsu.ox.ac.uk/crystal/field.cgi?id=20084
20084_475	Vitamin A	http://biobank.ctsu.ox.ac.uk/crystal/field.cgi?id=20084
20084_476	Vitamin B6	http://biobank.ctsu.ox.ac.uk/crystal/field.cgi?id=20084
20084_477	Vitamin B12	http://biobank.ctsu.ox.ac.uk/crystal/field.cgi?id=20084
20084_478	Vitamin C	http://biobank.ctsu.ox.ac.uk/crystal/field.cgi?id=20084
20084_479	Vitamin D	http://biobank.ctsu.ox.ac.uk/crystal/field.cgi?id=20084
20084_480	Vitamin E	http://biobank.ctsu.ox.ac.uk/crystal/field.cgi?id=20084
20084_481	Folic acid	http://biobank.ctsu.ox.ac.uk/crystal/field.cgi?id=20084
20084_482	Chromium	http://biobank.ctsu.ox.ac.uk/crystal/field.cgi?id=20084
20084_483	Magnesium	http://biobank.ctsu.ox.ac.uk/crystal/field.cgi?id=20084
20084_484	selenium	http://biobank.ctsu.ox.ac.uk/crystal/field.cgi?id=20084
20084_485	Calcium	http://biobank.ctsu.ox.ac.uk/crystal/field.cgi?id=20084
20084_486	Iron	http://biobank.ctsu.ox.ac.uk/crystal/field.cgi?id=20084
20084_487	Zinc	http://biobank.ctsu.ox.ac.uk/crystal/field.cgi?id=20084
**GWAS ID**	**Outcome**	**Data showcase link**
J10_VIRALPNEUMO	Viral Pneumonia	https://risteys.finngen.fi/endpoints/J10_VIRALPNEUMO

Table S2. Detailed information of SNP selection.

GWAS ID	exposure	outcome	nsnp after significant threshold	nsnp after LD clump	nsnp after harmonization
20084_473	Glucosamine/chondroitin	viral pneumonia	31	5	4
20084_475	Vitamin A		1879	6	4
20084_476	Vitamin B6		1233	5	4
20084_477	Vitamin B12		1293	7	7
20084_478	Vitamin C		170	4	2
20084_479	Vitamin D		1404	5	5
20084_480	Vitamin E		1603	10	8
20084_481	Folic acid		1845	5	4
20084_482	Chromium		2707	29	28
20084_483	Magnesium		1619	9	7
20084_484	Selenium		1548	9	9
20084_485	Calcium		143	9	7
20084_486	Iron		1562	9	9
20084_487	Zinc		1416	6	6

Table S3. Information for Pleiotropy and Heterogeneity test

GWAS ID	Exposure	Outcome	Pleiotropy		Heterogeneity	
			Method	*p*-value	Method	*p*-value
20084_476	Vitamin B6	viral pneumonia	chochran Q	0.96	Egger Intercept	0.82
20084_484	Selenium		chochran Q	0.92	Egger Intercept	0.79

A

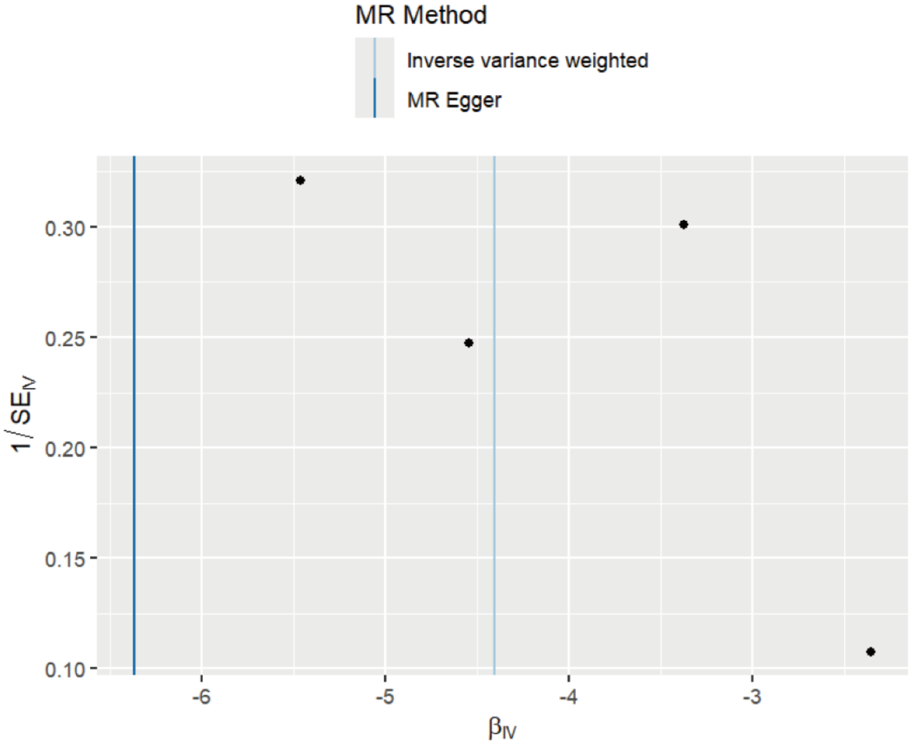

B

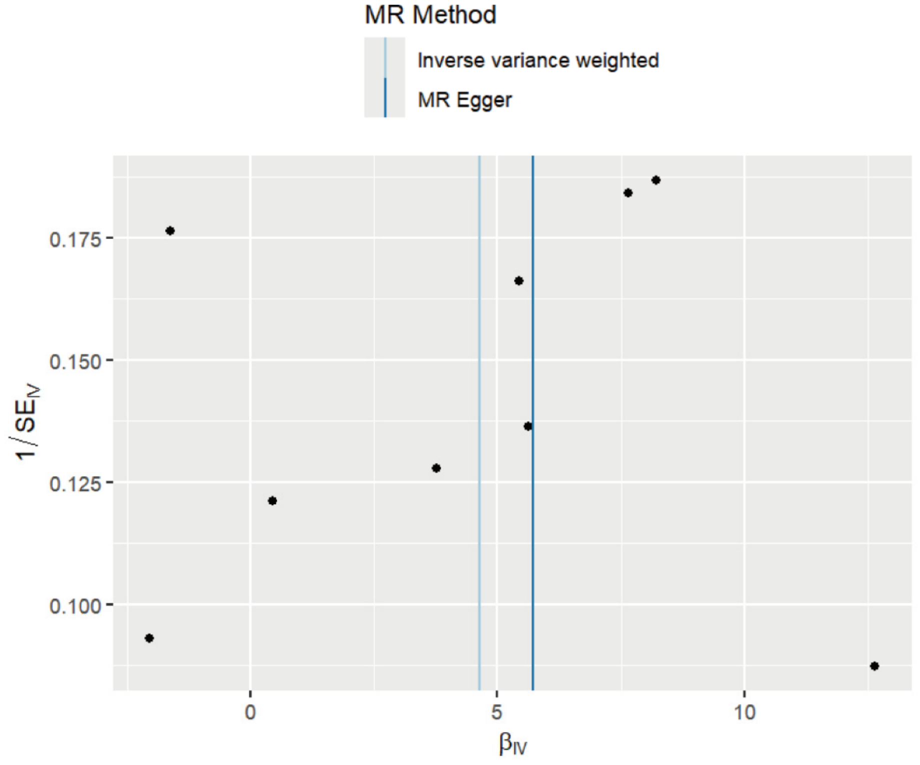

Figure S1 Funnel plots of SNPs used for analysis the casual effects of

Vitamin B6 and Selenium on viral pneumonia. (A) Funnel plot of Vitamin B6's effect on viral pneumonia. (B) Funnel plot of Selenium's effect on viral pneumonia.
